# Predictive Value of the Pretreatment Neutrophil-to-Lymphocyte Ratio in Head and Neck Squamous Cell Carcinoma

**DOI:** 10.3390/jcm7100294

**Published:** 2018-09-20

**Authors:** Miao-Fen Chen, Ming-Shao Tsai, Wen-Cheng Chen, Ping-Tsung Chen

**Affiliations:** 1 Department of Radiation Oncology, Chang Gung Memorial Hospital, Chiayi 61363, Taiwan; rto_chen@yahoo.com.tw; 2 Chang Gung University College of Medicine, Taoyuan 33302, Taiwan; b87401061@cgmh.org.tw (M.-S.T.); chencgmh@gmail.com (P.-T.C.); 3 Department of Otolaryngology & Head and Neck Surgery, Chang Gung Memorial Hospital, Chiayi 61363, Taiwan; 4 Department of Hematology and Oncology, Chang Gung Memorial Hospital, Chiayi 61363, Taiwan

**Keywords:** head and neck squamous cell carcinoma (HNSCC), neutrophil-to-lymphocyte ratio (NLR), myeloid-derived suppressor cells (MDSC), aldehyde dehydrogenase 1 (ALDH1), prognosis

## Abstract

This study assessed the significance of the neutrophil-to-lymphocyte ratio (NLR) in head and neck squamous cell carcinoma (HNSCC), and the relationships of the NLR with the aldehyde dehydrogenase 1 (ALDH1) level in tumors and the proportion of myeloid-derived suppressor cells (MDSCs) in the peripheral circulation. In total, 227 HNSCC patients who had received curative treatment at our hospital were enrolled into the present study. The NLR of each HNSCC patient before treatment was calculated. The associations of NLR with various clinicopathological parameters and prognoses were then examined. In addition, correlations between the proportion of MDSCs and level of ALDH1 with the NLR were assessed. Our data revealed that an elevated NLR was significantly correlated with the risk of developing locoregional recurrence and with a reduced overall survival in HNSCC patients. Multivariate analyses revealed that the NLR pretreatment and surgical resection were significantly correlated with the rate of treatment failure and the overall survival rate in HNSCC patients. Furthermore, the levels of ALDH1 in tumors and MDSCs in the peripheral circulation were significantly correlated with the prognosis of HNSCC, and the NLR was positively correlated with MDSC levels in the circulation and ALDH1 staining intensity in tumor specimens. In conclusion, the NLR has power in predicting the expression of ALDH1 in tumors, the circulating level of MDSCs, and the prognosis in HNSCC. We suggest that the NLR is an important biomarker that can assist the clinician and patient in making informed decisions regarding treatment options for HNSCC patients.

## 1. Introduction

Head and neck squamous cell carcinoma (HNSCC) is a heterogeneous disease occurring in various sites, including the oral cavity, oropharynx, and hypopharynx [1]. Treatment failure and locoregional recurrence are common and account for the majority of deaths [2]. Identification of potential molecular markers predicting aggressive tumor growth and treatment response is important for the effective management and prognosis of HNSCC.

Abundant epidemiological data have revealed a strong correlation between inflammation and cancer incidence. Systemic inflammation is a recognized characteristic of malignancy, and numerous inflammatory markers have been investigated as prognostic indicators for cancer patients [3,4]. Host inflammatory responses were reported to play an important role in tumor development and progression [5]. The neutrophil-to-lymphocyte ratio (NLR) is an inflammatory- and immunologically-based index [6,7]. The NLR may reflect host inflammatory responses and changes in the tumor microenvironment [8]. An elevated NLR in many solid tumors, including HNSCC, has been associated with reduced survival [8,9,10]. However, the predictive value of the NLR in the immune and treatment responses of HNSCC is still unclear. Myeloid-derived suppressor cells (MDSCs) have been reported to attenuate immune surveillance and induce an immunosuppressive tumor microenvironment to promote cancer metastasis [11,12]. We previously reported that the recruitment of MDSCs is significantly associated with a poor prognosis in patients with HNSCC [13]. Furthermore, one of the main causes of treatment failure is the emergence of resistant cancer cells after therapy, which can be partly explained by cancer stem cells (CSCs) [14,15]. CSCs produce immunosuppressive molecules that attenuate the immune system and recruit or activate cells that suppress the immune system, such as MDSCs [16,17,18]. Aldehyde dehydrogenase 1 (ALDH1), a novel CSC-like cell marker, was reported to play important roles in the treatment response and tumor-promoting microenvironment in squamous cell carcinomas (SCCs) of the aerodigestive tract [19,20,21]. Accordingly, in the present study, we examined the predictive role of an elevated NLR in the prognosis and relationships of the NLR with the ALDH1 level in tumors, and the proportion of MDSCs in the peripheral circulation of patients with HNSCC.

## 2. Materials and Methods 

### 2.1. Study Population and Study Design

The study protocol was approved by the institutional review board of Chang Gung Memorial hospital (No. 1035434B). Written informed consent was obtained from all patients. A total of 227 patients with a histologically confirmed diagnosis of HNSCC who received curative treatment were enrolled in our study. The planned treatments included definitive radiotherapy and chemotherapy (CCRT) or surgery +/− adjuvant treatment for patients with HNSCC, according to the guidelines proposed by the oncology team at our hospital. We included patient demographics (age and sex), diagnosis, clinical stage, and treatment characteristics (Table 1). In addition, the patients enrolled in the study had available data regarding ALDH1 levels in tumor specimens and/or the percentage of MDSCs in the peripheral circulation. The NLR was calculated by dividing the absolute neutrophil count by the absolute lymphocyte count. The mean absolute neutrophil count was 5.77 ± 3.29 × 10^3^/µL, and the mean absolute lymphocyte count was 1.55 ± 0.85 × 10^3^/µL. To assess the predictive value of the NLR, NLR was redefined as a binary variable by finding the value from a receiver operating characteristic (ROC) curve that maximized the percentage correctly classified for predicting tumor recurrence after treatment. The optimal cut-offs for NLR was 3. Accordingly, all HNSCC patients were divided into two groups according to the pretreatment NLR: high (NLR ≥ 3) and low (NLR < 3) groups.

### 2.2. Immunohistochemical (IHC) Staining

Formalin-fixed and paraffin-embedded tissues, collected at diagnosis from 227 patients with HNSCC who had completed curative treatment, were subjected to IHC analysis (Table 2). The IHC data were assessed using the semiquantitative immunoreactive score, and positive staining was defined as an immunoreactive score ≥ 2 [21]. The clinical end points were overall survival (OS), disease-free survival, and failure pattern. Disease failure was defined as documented locoregional recurrence and/or distant metastases.

### 2.3. MDSC Isolation and Flow Cytometry Analysis 

Peripheral blood samples were obtained from 118 patients with pathologically and clinically confirmed HNSCC (Table 3). To assess the proportion of MDSCs among peripheral blood mononuclear cells (PBMCs), multicolor fluorescence-activated cell sorting (FACS) was performed using the FACS Caliber flow cytometer (BD Biosciences, San Jose, CA, USA). Human low-density neutrophils and granulocytic MDSCs are closely related, and presently there is no generally accepted consensus on mutually exclusive definitions for these cell types [22]. In the majority of oncological studies, human granulocytic MDSCs are characterized as CD14^−^CD15^+^CD11b^+^HLA-DR^−^ cells [7]. Accordingly, the human MDSC subset characterized as CD11b^+^CD14^−^HLA-DR^−^ cells was sorted from the peripheral blood. The leukocytes were separated from the peripheral blood using a Ficoll gradient before analysis or sorting. Multicolor cell analysis was performed using the following antibodies: PerCP-Cy5.5-conjugated CD14, polyethylene (PE)-conjugated CD11b, and fluorescein isothiocyanate-conjugated HLA-DR. The percentage of MDSCs was measured using multicolor flow cytometry, and isotype-specific antibodies were used as negative controls.

### 2.4. Statistical Analysis

The Kaplan–Meier method was used to plot survival curves, and the log-rank test was used to determine differences in the survival curves between the two groups. The Cox proportional hazard model was used to compute hazard ratios with 95% confidence intervals (CI) after adjustment for esophageal cancer treatment and clinical characteristics. All analyses were conducted using SAS statistical software, version 9.2 (SAS Institute, Cary, NC, USA).

## 3. Results

### 3.1. Correlations Between the Pretreatment NLR and Clinicopathological Characteristics of HNSCC Patients

**Figure 1:**
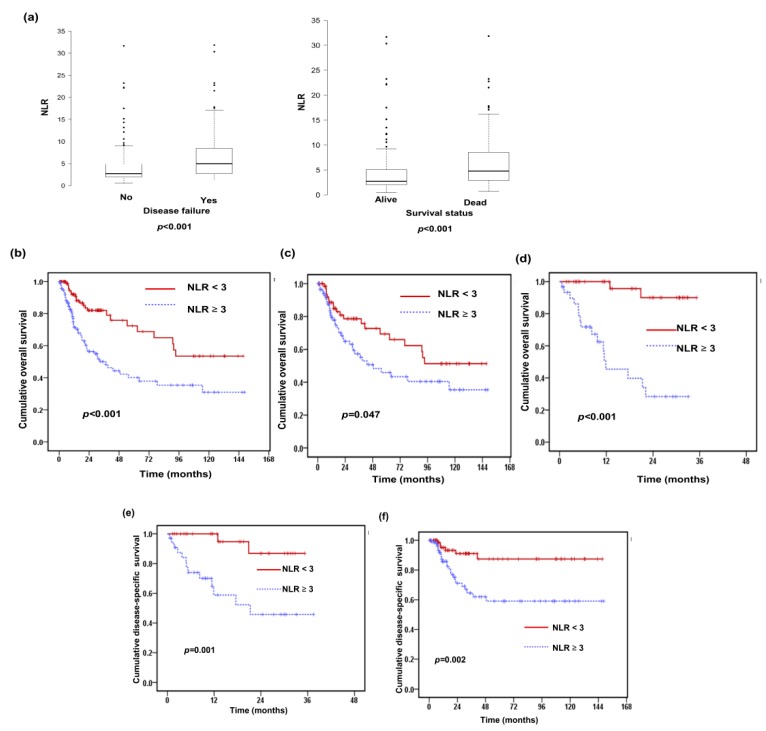
Correlations between the baseline neutrophil-to-lymphocyte ratio (NLR) and the prognosis for patients with head and neck squamous cell carcinoma (HNSCC). (**a**) The pretreatment NLR in HNSCC patients. Box-plot showing NLR at baseline was elevated in patients with locoregional recurrence and having higher risk of death during follow-up. The data showed the third quartile (Q3) and first quartile (Q1) range of the data and data outliers. Lines indicate the median values. The survival differences are according to the pre-treatment NLR (NLR ≥ 3 vs. NLR < 3) in (**b**) all, (**c**) subgroup of oral cancer patients, and (**d**) the subgroup of patients with oro-hypopharyngeal cancer. In addition, the differences of disease-specific survival (DSS) are according to the pre-treatment NLR in (**e**) the subgroup of definite CCRT, and (**f**) the subgroup of surgery.

A total of 227 patients with HNSCC were enrolled in this study (Table 1). The median follow-up time was 25.6 months (range 1.37–148 months). There were 114 (50%) patients with clinical tumor stage T1/T2 disease and 140 (62%) with clinical lymph node involvement. Of these patients, 162 (71%) received surgery with or without adjuvant treatment, and the others received definitive RT and chemotherapy. The pretreatment NLR was calculated as the ratio of the absolute neutrophil count to the lymphocyte count. The median pretreatment NLR of the overall cohort was 3.12 (range 0.5 to 31.8). At baseline, 119 (52%) patients had a high NLR of three or higher and 108 (48%) a low NLR less than three. The relationships between the clinicopathological variables and the pretreatment NLR values are shown in Table 1 and Figure 1a. A high NLR at baseline was significantly associated with locoregional recurrence (*p* < 0.001) and a higher risk of death during follow-up (*p* < 0.001). To further examine whether the pretreatment NLR was associated with the outcomes of HNSCC patients after curative treatment, Kaplan–Meier survival analysis was used to compare the low and high NLR subgroups. Patients with a high pretreatment NLR had a shorter overall survival (OS) time (*p* < 0.001; Figure 1b). As shown in Figure 1c,d, a high NLR was significantly associated with a reduced OS rate in both oro/hypopharyngeal cancer (*p* < 0.001) and oral cancer patients (*p* = 0.047). The results of multivariate analyses (Table 4 and Table 5) revealed that the pretreatment NLR and surgical resection were significantly correlated with the risk of developing disease failure after treatment and with the OS rate. We further analyzed the predictive value of the NLR according to treatment modality. The data revealed that high NLR was associated with shorter disease-specific survival (DSS) time in patients treated with CCRT and those treated with surgery (Figure 1e,f). Moreover, in the subgroup of surgery, a high NLR was the significant predictor independent of clinical T-stage. In the subgroup of CCRT, a high NLR was associated with shorter disease-specific survival time in patients with advanced tumor stage (*p =* 0.021), but not in those with early tumor stage (*p =* 0.053).

### 3.2. Relationships of ALDH1 Expression with the Pretreatment NLR and Clinical Outcome

**Figure 2:**
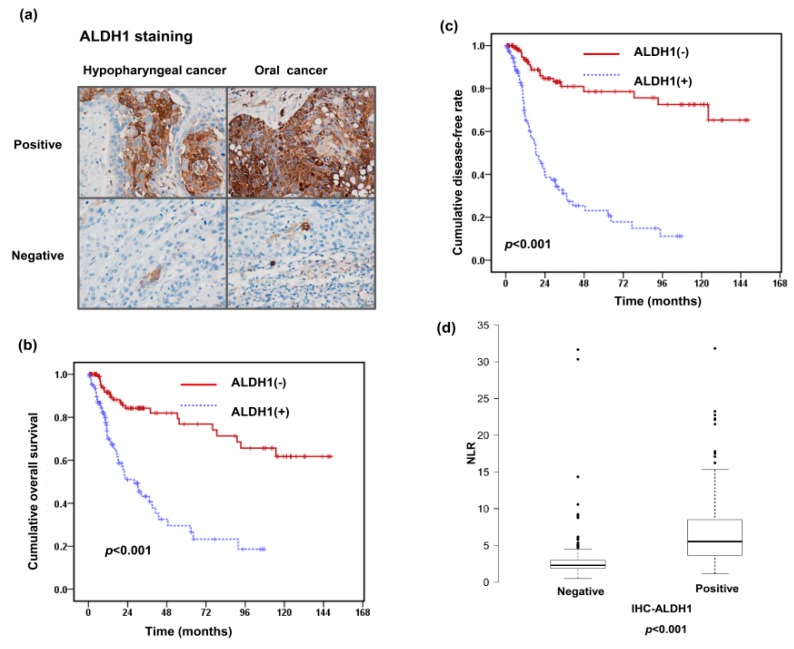
Relationships between NLR, aldehyde dehydrogenase 1 (ALDH1) expression level, and clinical outcome. (**a**) Representative images of immunohistochemical (IHC) staining with anti-ALDH1 antibodies of oral cancer and hypopharyngeal cancer specimens. Survival differences demonstrated according to the staining of ALDH1 in (**b**) overall survival rate and (**c**) disease failure-free rate. (**d**) NLR levels in the groups of HNSCC patients with and without ALDH1 positive staining in tumor specimens. The data show the third quartile (Q3) and first quartile (Q1) range of the data and data outlier. Lines indicate the median values.

We previously reported that positive ALDH1 staining was significantly related to a poor treatment response and higher disease failure rate in oral squamous cell carcinoma (SCC) [21]. Accordingly, we analyzed the predictive role of ALDH1 levels in the clinical outcome and its correlation with the pretreatment NLR in the 227 HNSCC patients. Figure 2a shows representative slides of positive and negative ALDH1 staining in HNSCC specimens at diagnosis. IHC revealed ALDH1 overexpression in 109 (48%) in these patients. As shown in Table 2, positive ALDH1 staining was significantly associated with the risk of lymph node involvement (*p* = 0.016), a higher rate of locoregional failure (*p* < 0.001), and distant metastasis (*p* = 0.019). As shown in Figure 2b,c, positive staining of ALDH1 was significantly associated with a higher locoregional failure rate and lower OS rate. In the multivariate analysis, positive staining of ALDH1 was significantly associated with a higher risk of developing disease failure and a shorter OS time in HNSCC (Table 6 and Table 7). Furthermore, the distribution of the pretreatment NLR was significantly associated with ALDH1 staining in tumor specimens (Table 2 and Figure 2d). Based on the results, we suggest that positive staining of ALDH1 is an independent predictor of shorter survival and a higher rate of disease failure, and a high pretreatment NLR plays a role in predicting ALDH1 expression levels and subsequently a poor prognosis in HNSCC.

### 3.3. Predictive Role of Pretreatment NLR on Levels of CD11b^+^CD14^−^HLA-DR^−^ Cells in Peripheral Circulation

**Figure 3:**
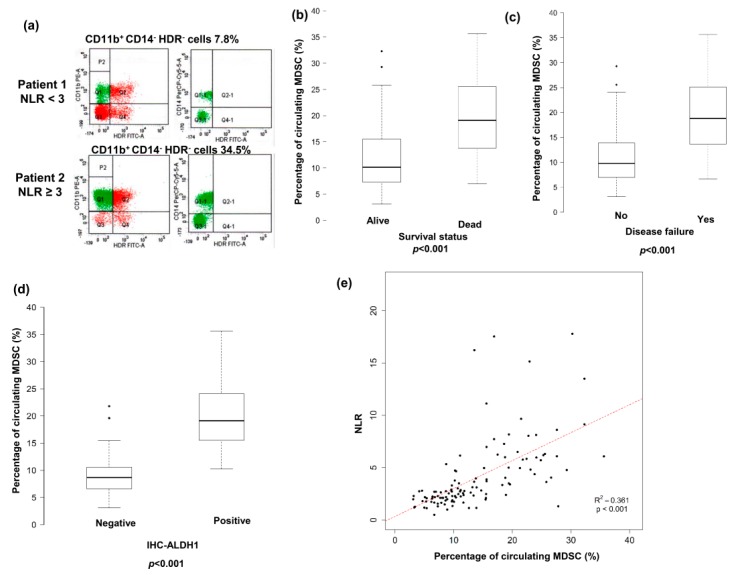
Correlation between pre-treatment NLR in the levels of circulating CD11b^+^CD14^-^HLA-DR^−^ cells and ALDH1. (**a**) Flow cytometric analysis of circulating CD11b^+^CD14^-^HLA-DR^−^ cells in isolated peripheral blood mononuclear cells (PBMCs). HLA-DR−CD11b+ cells were gated, and the CD14 negative population was then selected. Representative data from two cancer patients are shown (upper row, the patient with pretreatment NLR < 3; lower row, the patient with pretreatment NLR ≥ 3). Elevated circulating levels of CD11b^+^CD14^-^HLA-DR^−^ cells associated with the higher risk of death (**b**), disease recurrence after treatment (**c**), and ALDH1 positive staining (**d**). (**e**) Positive correlation between the levels of CD11b^+^CD14^−^HLA-DR^−^ cells and pre-treatment NLR in the peripheral circulation.

Accumulating evidence indicates that MDSCs, a population of cells with suppressive activity, contribute to the negative regulation of immune responses and subsequently to tumor promotion [11]. We previously reported that circulating MDSC levels were significantly increased in patients with HNSCC, and this was associated with the clinical tumor burden [13]. In the present study, the percentage of CD11b^+^CD14^−^HLA-DR^−^ cells, a subset of MDSCs, in the peripheral circulation of 118 patients with HNSCC was evaluated by flow cytometry. Representative flow cytometry data from two HNSCC patients are shown in Figure 3a. The mean percentage of CD11b^+^CD14^−^HLA-DR^−^ cells in the peripheral blood mononuclear cells of the 118 HSCC patients was 11.6 ± 7.4%. As shown Figure 3b,c, the percentage of CD11b^+^CD14^−^HLA-DR^−^ cells was significantly correlated with the risk of developing disease failure and death after treatment (*p* < 0.001). An increased MDSC level was reported to be associated with attenuating immune surveillance noted in CSC tumors [16,17,21]. Figure 3d shows that the level of MDSCs was significantly higher in the ALDH1-positive group than in the ALDH1-negative group (*p* < 0.001). The 118 patients were further divided into two groups according to the mean CD11b^+^CD14^−^HLA-DR^−^ cell percentage at diagnosis (11.6%): high (≥11.6%) and low (<11.6%) groups. As shown in Table 3, a high percentage of CD11b^+^CD14^−^HLA-DR^−^ cells was associated with a more advanced clinical tumor stage (T3/T4, *p* = 0.005), lymph node involvement (*p* = 0.018), a higher pretreatment NLR, and shorter survival compared with a low CD11b^+^CD14^−^HLA-DR^−^ cell percentage. In the multivariate analysis, a higher percentage of circulating MDSCs was significantly associated with a higher risk of developing disease failure and a shorter survival in patients with HNSCC (Table 8 and Table 9). We further assessed the usefulness of the NLR in predicting the CD11b^+^CD14^−^HLA-DR^−^ cell percentage. A strong correlation was found between the pretreatment NLR and the percentage of CD11b^+^CD14^−^HLA-DR^−^ cells in peripheral circulation of HNSCC patients (Figure 3e).

## 4. Discussion

The tumor microenvironment plays an important role in cancer development and progression and may be associated with systemic inflammation [23]. Neutrophils form the first line of host immune defense against bacterial and fungal infections [24]. Compared with their role in host defenses, which is relatively well established, we are just beginning to learn about the precise role of neutrophils in cancer [25,26]. Many recent studies suggested that an elevated NLR is associated with poor survival of subjects with cancer [8,27], including head and neck cancer [10,28]. In the present study, an advantage of our analyses was that the results were based on a relatively large population of HNSCC patients from a single institute, with available information regarding staging and primary treatment details. Based on the analyses of 227 HNSCC patients who received curative treatment, an elevated pretreatment NLR was significantly associated with higher loco-regional recurrence rate and reduced OS rate. According to univariate and multivariate analyses, a pretreatment NLR of three or higher was associated with a shorter OS compared with a NLR below three. Treatment policy included surgery with or without adjuvant treatment for oral cancers and definitive radiation and chemotherapy for oropharyngeal and hypopharyngeal cancers. We further analyzed the predictive value of the NLR according to treatment modality. The data revealed that an increased NLR was a significant predictor for poor prognosis in patients treated with CCRT and those treated with surgery. Based on these results, the NLR is a useful baseline variable for assessing prognosis in HNSCC patients considered for curative treatment. 

Circulating blood contains several types of immune cells that participate in the immune response. The interactions among the various populations of immune cells have been recognized as critical in forming the immune microenvironment, which provides the milieu for the anti-cancer immune response to occur [29,30,31,32]. MDSCs constitute an immature population of myeloid cells thought to be an important subset of cells that contribute to an immunosuppressive tumor microenvironment, and MDSC numbers are significantly increased in cancer patients [33,34]. Increasing evidence has demonstrated an association between suppressive neutrophils and granulocytic MDSCs related to immune suppression and their relevance to disease [35]. Many of the pro-tumor features of suppressive neutrophils are shared with granulocytic MDSCs, and the distinction between these two cell populations is a matter of intensive debate. We previously found that MDSC recruitment provided a microenvironment conducive to tumor growth and the development of treatment resistance in HNSCC [13]. To date, granulocytic MDSCs have been defined mainly as CD11b^+^CD14^−^HLA-DR^−^ cell lineages in human cancers [7,22,35]. Accordingly, we characterized the proportions of CD11b^+^CD14^−^HLA-DR^−^ myeloid cells in a cohort of HNSCC patients. FACS analyses revealed that the percentage of these MDSCs was correlated with the clinical tumor burden, disease status, and survival. We further demonstrated a positive correlation between the NLR and circulating CD11b^+^CD14^−^HLA-DR^−^ cell level in HNSCC patients. In the present study, we showed that the pretreatment NLR was related to the circulating CD11b^+^CD14^−^HLA-DR^−^ cell level and disease progression.

Much of the relationship between immune cells, either circulating or within tumors, and disease outcome in cancer can probably be explained by the inflammatory response that is secondary to the cancer. CSCs are becoming recognized as being responsible for metastasis and treatment resistance [36,37]. ALDH1, a detoxifying enzyme, has been identified as a novel CSC-like cell marker and is relevant to the prognosis of cancers [19,20,38,39]. Immune evasion was reported to play a role in the contribution of CSCs in tumor promotion [16,40]. CSCs can recruit cells that suppress the immune system, such as the activation of myeloid-derived suppressor cells (MDSCs), to attenuate immune surveillance [16,17,18,41,42,43]. Our previous data revealed correlations between ALDH1 expression levels and treatment resistance, CSC-like properties, higher circulating MDSC levels, and poor prognosis in oral SCC. Accordingly, we further examined the predictive value of ALDH1 for HNSCC prognosis and the correlations of the ALDH1 level with the MDSC level and NLR. By analyzing the clinical outcomes of 227 patients with HNSCC, the elevated expression of ALDH1 was correlated with a higher incidence of lymph node involvement, higher disease failure rate, and lower survival rate. Moreover, there were significant correlations among ALDH1 IHC staining, the levels of circulating MDSCs, and NLR. Patients with a higher NLR had a higher ALDH1 level in their tumors and more MDSCs in the peripheral circulation, which are associated with poor prognosis of HNSCC. The current study is limited by the inherent nature of investigating a hospital-based registry and the nonrandomized approach to treatment selection. Furthermore, we could not account for potential unmeasured selection biases regarding performance status, comorbidity, access to health care, or other patient-related factors.

## 5. Conclusions

With the increasing use of personalized therapy, patient selection has become an important issue in assessing efficacy. The targeted therapy of CSCs may enhance the treatment response, thereby resulting in improved clinical outcomes in patients with HNSCC. In addition, MDSCs have been suggested to be a novel target for multiple cancers, and numerous clinically available agents have been developed [34]. Thus, it is imperative to identify clinically feasible parameters highly relevant to the characteristics of CSC and the level of MDSCs. The NLR is a cheaper and faster laboratory measure compared with other biomarkers, and it does not involve any additional cost. In the present study, we showed that the NLR was relevant to ALDH1 and MDSC levels and a strong prognostic indicator for HNSCC patients. Discussions based on pretreatment NLR results may help the patient decide whether the side effects of curative treatments are worth the risk. We suggest the NLR to be an important biomarker for patients that can assist the clinician and patient to make informed decisions regarding treatment options.

## Figures and Tables

**Table 1:** Characteristics of head and neck squamous cell carcinoma (HNSCC) patients with curative-intent treatment.

	Number of Patients		
	NLR < 3	NLR ≥ 3	*p-*Value
Patient	108	119	
Age			
<55 (median)	52	53	0.58
≥55	56	66	
Differentiation			0.38
Well differentiated	39	39	
Moderately differentiated	38	41	
Poorly differentiated	23	32	
Unknown	8	7	
Tumor stage			0.073
≤T2	61	53	
T3–T4	47	66	
Clinical LN involvement			0.87
Negative	42	45	
Positive	66	74	
Tx policy			0.98
Definite CCRT	31	34	
Surgery +/− neoadjuvant/adjuvant Tx	77	85	
Location			0.23
Oral cavity	72	88	
Pharynx (Oro-Hypo)	36	31	
Loco-regional recurrence			<0.001 *
Control	88	65	
Failure	20	54	
Distant metastasis			0.146
Negative	102	108	
Positive	6	13	
Status			<0.001 *
Alive	86	66	
Dead	22	53	

NLR: neutrophil-to-lymphocyte ratio; CCRT: radiotherapy and chemotherapy; LN: lymph node; Tx: treatment; *: Statistically significant covariate; T: tumor.

**Table 2:** Characteristics of head and neck squamous cell carcinoma (HNSCC) patients with curative-intent treatment.

	Number of Patients		
	ALDH1 (−)	ALDH1 (+)	*p-*Value
Patients	118	109	
Age			
<55 (median)	56	49	0.71
≥55	62	60	
Differentiation			0.38
Well differentiated	44	34	
Moderately differentiated	39	40	
Poorly differentiated	27	28	
Unknown	8	7	
Tumor stage			0.13
≤T2	65	49	
T3–T4	53	60	
Clinical LN involvement			0.016 *
Negative	54	33	
Positive	64	76	
Tx policy			0.42
Definite CCRT	35	34	
Surgery +/− neoadjuvant/adjuvant Tx	83	75	
Location			0.96
Oral cavity	72	77	
Pharynx (Oro-Hypo)	36	32	
NLR			<0.001 *
<3	89	19	
≥3	29	90	
Loco-regional recurrence			<0.001 *
Control	104	49	
Failure	14	60	
Distant metastasis			0.019 *
Negative	113	95	
Positive	5	14	
Status			<0.001 *
Alive	96	56	
Dead	22	53	

ALDH1. aldehyde dehydrogenase 1; *: Statistically significant covariate.

**Table 3:** Characteristics of head and neck squamous cell carcinoma (HNSCC) patients with curative-intent treatment.

	Number of Patients		
	MDSC (Low)	MDSC (High)	*p-*Value
Patients	59	59	
Age			
<55 (median)	28	26	0.72
≥55	31	33	
Differentiation			0.15
Well differentiated	12	9	
Moderately differentiated	24	18	
Poorly differentiated	17	23	
Unknown	6	9	
Tumor stage			0.005 *
≤T2	35	20	
T3–T4	24	39	
Clinical LN involvement			0.018 *
Negative	25	13	
Positive	34	46	
Tx policy			0.016*
Definite CCRT	26	39	
Surgery +/− neoadjuvant/adjuvant Tx	33	20	
Location			0.19
Oral cavity	29	22	
Pharynx (Oro-Hypo)	30	37	
NLR			<0.001 *
<3	51	13	
≥3	8	46	
ALDH1 staining			<0.001 *
Negative	55	9	
Positive	4	50	
Disease failure			<0.001 *
Negative	52	26	
Positive	7	33	
Status			<0.001 *
Alive	54	34	
Dead	5	25	

MDSC: myeloid-derived suppressor cell; *: Statistically significant covariate.

**Table 4:** Adjusted hazard ratio (HR) of determining factors associated with overall survival (OS) of patients with head and neck squamous cell carcinoma (HNSCC).

Variable	HR	95% CI	*p*-Value
Age			
<50	Ref		
≥50	1.51	0.92–2.47	0.1
Differentiation			
Well–Moderately differentiated	Ref		
Poorly differentiated.	0.77	0.43–1.36	0.36
Clinical T stage			
≥T2	Ref		
T3–T4	0.85	0.54–1.36	0.5
Clinical N stage			
N 0	Ref		
N (+)	1.49	0.9–2.47	0.12
NLR			
<3	Ref		
≥3	2.69	1.62–4.46	<0.001 *
Treatment			
Definite CCRT	Ref		
Surgery +/− neoadjuvant/adjuvant Tx	0.44	0.24–0.79	0.006 *

CI: Confidence Interval; Ref: Reference Group; N: Lymph node staging; *: Statistically significant covariate.

**Table 5:** Adjusted hazard ratio (HR) of determining factors associated with disease failure of patients with head and neck squamous cell carcinoma (HNSCC).

Variable	HR	95% CI	*p*-Value
Age			
<50	Ref		
≥50	1.26	0.8–1.99	0.314
Differentiation			
Well–Moderately differentiated	Ref		
Poorly differentiated	0.87	0.52–0.47	0.61
Clinical T stage			
≤T2	Ref		
T3–T4	0.94	0.61–1.45	0.77
Clinical N stage			
N 0	Ref		
N (+)	1.6	0.99–2.58	0.057
NLR			
< 3	Ref		
≥ 3	2.71	1.69–4.38	<0.001 *
Treatment			
Definite CCRT	Ref		
Surgery +/− neoadjuvant/adjuvant Tx	0.37	0.22–0.63	<0.001 *

*: Statistically significant covariate.

**Table 6:** Adjusted hazard ratio (HR) of determining factors associated with overall survival (OS) of patients with head and neck squamous cell carcinoma (HNSCC).

Variable	HR	95% CI	*p-*Value
Age			
<50	Ref		
≥50	1.36	0.84–2.12	0.21
Differentiation			
Well–Moderately differentiated	Ref		
Poorly differentiated	0.79	0.45–1.39	0.42
Clinical T stage			
≤T2	Ref		
T3–T4	1.01	0.62–1.60	0.98
Clinical N stage			
N 0	Ref		
N (+)	1.17	0.71–1.93	0.53
ALDH1			
Negative	Ref		
Positive	4.1	2.43–6.92	<0.001 *
Treatment			
Definite CCRT	Ref		
Surgery +/− neoadjuvant/adjuvant Tx	0.48	0.27–0.87	0.015 *

*: Statistically significant covariate.

**Table 7:** Adjusted hazard ratio (HR) of determining factors associated with disease failure of patients with head and neck squamous cell carcinoma (HNSCC).

Variable	HR	95% CI	*p*-Value
Age			
<50	Ref		
≥50	1.12	0.71–1.77	0.62
Differentiation			
Well–Moderately differentiated	Ref		
Poorly differentiated	0.88	0.53–1.47	0.63
Clinical T stage			
≤T2	Ref		
T3–T4	1.1	0.70–1.71	0.68
Clinical N stage			
N 0	Ref		
N (+)	1.26	0.78–2.03	0.35
ALDH1			
Negative	Ref		
Positive	5.92	3.46–10.11	<0.001 *
Treatment			
Definite CCRT	Ref		
Surgery +/− neoadjuvant/adjuvant Tx	0.42	0.25–0.71	0.001 *

*: Statistically significant covariate.

**Table 8:** Adjusted hazard ratio (HR) of determining factors associated with overall survival (OS) of patients with neck squamous cell carcinoma (HNSCC).

Variable	HR	95% CI	*p*-Value
Age			
<50	Ref		
≥50	0.71	0.32–1.55	0.39
Differentiation			
Well–Moderately differentiated	Ref		
Poorly differentiated	0.44	0.18–1.06	0.07
Clinical T stage			
≤T2	Ref		
T3–T4	1.04	0.44–2.48	0.93
Clinical N stage			
N 0	Ref		
N (+)	1.19	0.49–2.90	0.70
MDSC			
Low	Ref		
High	6.19	2.34–18.0	<0.001 *
Treatment			
Definite CCRT	Ref		
Surgery +/− neoadjuvant/adjuvant Tx	0.43	0.19–0.98	0.044 *

*: Statistically significant covariate.

**Table 9:** Adjusted hazard ratio (HR) of determining factors associated with disease failure of patients with neck squamous cell carcinoma (HNSCC).

Variable	HR	95% CI	*p*-Value
Age			
<50	Ref		
≥50	0.70	0.34–1.40	0.31
Differentiation			
Well–Moderately differentiated	Ref		
Poorly differentiated	0.61	0.29–1.25	0.18
Clinical T stage			
≤T2	Ref		
T3–T4	1.28	0.60–2.76	0.52
Clinical N stage			
N 0	Ref		
N (+)	1.59	0.68–3.75	0.29
ALDH1			
Negative	Ref		
Positive	5.31	2.23–12.62	<0.001 *
Treatment			
Definite CCRT	Ref		
Surgery +/− neoadjuvant/adjuvant Tx	0.39	0.19–0.82	0.012 *

*: Statistically significant covariate.
